# Nutritional and Other Trace Elements and Their Associations in Raw King Bolete Mushrooms, *Boletus edulis*

**DOI:** 10.3390/ijerph19010417

**Published:** 2021-12-31

**Authors:** Jerzy Falandysz

**Affiliations:** Department of Toxicology, Faculty of Pharmacy, Medical University of Lodz, Muszyńskiego Street, 90-151 Łódź, Poland; jerzy.falandysz@umed.lodz.pl

**Keywords:** food toxicology, food composition, fungi, minerals, diet, organic food

## Abstract

The occurrence and associations of Ag, As, Ba, Bi, Cd, Co, Cu, Cs, Hg, Ni, Pb, Rb, Sb, Sr, Tl, U, V, W, and Zn, including data that have not been previously reported on Be, Hf, In, Li, Mo, Nb, Sn, Ta, Th, Ti and Zr, and the sum of (14) rare earth elements (ƩREE), were studied in a spatially diverse collection of the *B. edulis* caps, stipes, and whole fruiting bodies using a validated procedure with measurement by quadrupole ICP-MS. Toxic Cd and Pb were in *B. edulis* at concentrations below limits set by the European Union in regulations for raw cultivated mushrooms, while Ag, As, Hg, Sb, Tl, and U, which are not regulated, were at relatively low or typical levels as is usually found in mushrooms from an unpolluted area. The elements Be, Bi, Ga, Ge, Hf, In, Nb, Ta, Th, and W, and also ƩREEs, were found at relatively low concentrations in *B. edulis*, i.e., with levels from below 0.1 to below 0.01 mg kg^−1^ dw, and for Ʃ14 REEs, the median was 0.31 mg kg^−1^ dw. The composite samples of caps showed Ag, Cd, Cu, Cs, Ga, Ge, Hg, Mo, Ni, Rb, Sb, Ti, and Zn at higher concentrations than stipes, while Ba, Co, Hf, Sr, Tl, and Zr were found at higher concentrations in stipes than caps (*p* < 0.05). Mushrooms were characterized by a low coefficient of variation (CV) of below 20%, between sites for concentrations of As, Cu, Ge, Hg, Ni, V, and Zn, while substantial differences (CV > 100%) were found for Ba, Bi, Co, Hf, Zr, and ƩREEs, and an intermediate variation was found for Sr, W, and U. Principal component analysis performed on mushrooms allowed differentiation with respect to 13 collection sites and separation of a consignment that was specifically contaminated, possibly due to a legacy pollution, with significantly higher levels of Ba, Co, Ga, Li, Nb, Ni, Sr, Th, Ti, Y, Zr, and ƩREEs, and another due to possible recent pollution (Pb-gasoline and also Ni); two due to geological contamination because of the Bi, In, Sc, Sb, Sn, Ta, V and W; and one more, the Sudety Mts. site, which was considered as “geogenic/anthropogenic” due to Ag, As, Be, Cd, Cs, Ni, Pb, Rb, Tl, and U.

## 1. Introduction

Wild edible mushrooms are traditionally considered as having a value for human eaters and are a part of gourmet heritage worldwide [1,2]. The king bolete *Boletus edulis* Bull. is a symbiotic ectomycorrhizal species and has a tubular hymenophore. This mushroom is native to Europe and other regions in the northern hemisphere with a moderate climate [3]. It has been reported earlier as occurring widely also in North America, and recently some variants, e.g., *B. edulis* var. *grandedulis* (California king bolete), *Boletus rex-veris*, *B. regineus*, and *B. rubriceps*, have been identified there [4]. *Boletus bainiugan* is widely collected in Yunnan in southwestern China (earlier sometimes identified as *B. edulis*), whilst the correctly identified *B. edulis* species is more prevalent in the northeast of China and in the north-eastern region of the continental Asia [5]. *B. edulis* is a popular wild mushroom that is widely foraged by both recreational and commercial mushroomers. In 2014, 5212 tons of fresh wild mushrooms were harvested commercially in Poland, of which 795 tons were fungi species *B. edulis* [6]. *B. edulis* when fresh is considered a delicacy due to its sensory and anticipated nutritional values related to the high content of the element selenium (Se) and other antioxidants and bioactive compounds considered to have a positive health impact in humans [7,8,9,10]. When preserved, pickled, or dried, it is also prized, especially at the button stage of maturity [11,12,13]. *B. edulis* specimens at the mature developmental stage, if not consumed fresh (braised, fried, etc.), are usually dried, pickled/marinated, or soured (souring is a tradition associated with specific regions where locals have a good access to wild mushrooms) [11,12,14,15]. This species still flourishes in some parts of Poland.

In a crude estimation, mushrooms consist of around 90% water. Mushrooms for culinary purposes can be processed or preserved to extend their shelf-life, by, e.g., traditionally drying processes or, nowadays, by deep-freezing. When fresh or blanched, they are of low calorific value (energy from 100 g of dried *B. edulis* was approx. 407 ± 15 kcal) coming from the small amount of constitutional fatty acids (ƩFA): saturated, monounsaturated, and polyunsaturated present (in *B. edulis* ƩFA at 4.36% in dried caps and 1.75% in stipes and total fat in dried whole *B. edulis* at 8.51 ± 0.80%), total glucans (α-glucan +β-glucan) + d-glucose in oligosaccharides, sucrose and free d-glucose (in dried whole *B. edulis* at 31.0 ± 0.87%), and proteins (in dried whole *B. edulis* at 34.73 ± 2.31%) [11,12,16,17,18,19]. Processing and storage can have a substantial impact on the content and composition of chemical constituents of mushrooms including *B. edulis* [11,12].

Chitin, a structural polysaccharide polymer made of 2-(acetylamino)-2-deoxy-D-glucose units, with different chain lengths (mass) in different species, is a dietary fiber-like part of a mushroom cell wall that is hardly digested by human if at all. Humans have chitinases (chitotriosidase 1 and acid mammalian chitinase) and several chitinase-like proteins that can act against chitin-containing pathogens [20]. Chitin of fungi, like a dietary (plant) fiber, is considered as a desired constituent for smooth bowel function and good health.

As mentioned, *B. edulis* collected from the European regions are rich in Se, with mean concentrations in the range from 20 to 30 mg kg^−1^ dw [4,9,10,21,22,23]. Like other mushrooms, the *B. edulis* is also relatively rich in some macro elements including potassium (K) and phosphorous (P) and can be rich in magnesium (Mg), while it is relatively lacking in calcium (Ca) and sodium (Na) [24,25]. As previously reported, the median concentrations of K, P, Mg, Ca, and Na in caps of *B. edulis* were in the ranges: 20,000–38,000, 10,000–19,000, 680–930, 29–170, and 100–380 mg kg^−1^ dw, respectively [2,24]. The small amount of data available shows that crude *B. edulis* is also rich in sulphur (S), e.g., in the range from below 10,000 to 10,000 ± 3000 mg kg^−1^ dw (n = 2, Czech Republic) and 8300–11,000 mg kg^−1^ dw (n = 2, Finland) to 9080–13,870 mg kg^−1^ dw (n = 2, Canada) [26,27,28].

In *B. edulis* harvested away from local (anthropogenic) sources of emission, trace elements can be found both of a beneficial and toxic nature in terms of human health, i.e., essential, toxic, radiotoxic, or non-essential elements [15,29,30,31,32,33,34,35,36,37,38,39,40,41,42,43,44,45,46,47,48,49,50,51]. The artificial radioactive isotopes of caesium (^134^Cs and ^137^Cs) are bio-concentrated by ectomycorrhizal mushrooms and were found at high activity concentrations in the young, button maturity stage, fruiting bodies of *B. edulis* as well as *Amanita muscaria* many years after the major contamination events [52,53].

Knowledge about mineral constituents and contaminants of edible wild mushrooms is growing over the last decade, although gaps still exist [2]. Culinary preparation of fruiting bodies as food for humans have an effect on elements occurrence, while the factual data for a number of elements are limited or lacking [13,14,15,52]. This work aimed to fill some of these gaps and presents the results of a study that extends the range of data on Ag, As, Ba, Bi, Cd, Co, Cu, Cs, Hg, Ni, Pb, Rb, Sb, Sr, Tl, U, V, W, and Zn and also presents data where information was not previously available on Be, Hf, In, Li, Mo, Nb, Sn, Ta, Th, Ti, and Zr, including ƩREEs (the sum of 14 rare earth elements) in a large collection of the *B. edulis* fruiting bodies. The data were obtained using a validated procedure with measurement by quadrupole ICP-MS and principal component analysis (PCA) of associations [54,55,56]. The computer software Statistica version 13.1 (Statsoft, Inc., computer software) was used for statistical analysis.

## 2. Materials and Methods

**Figure 1:**
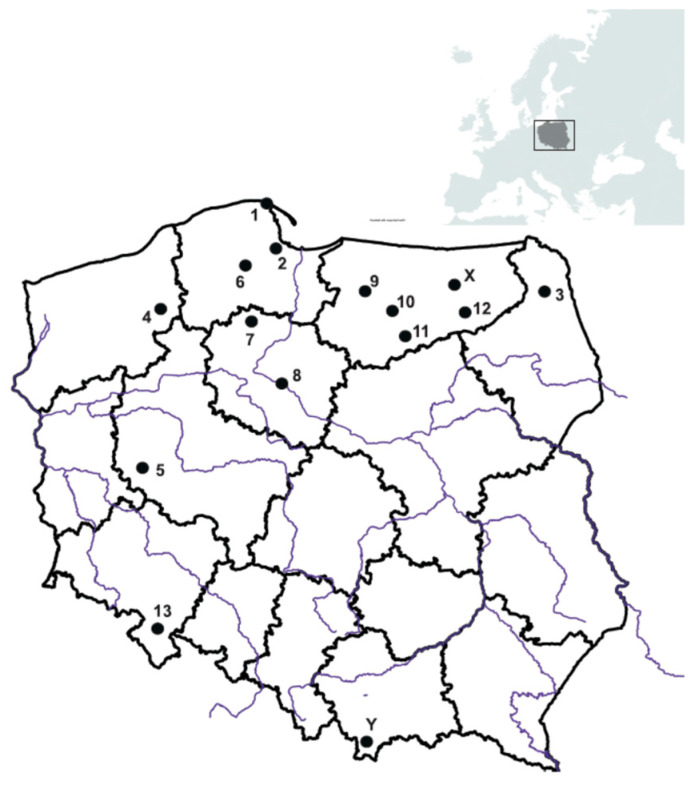
Sampling sites of *B. edulis* (1. Coastal Landscape Park; 2. Tricity Landscape Park, Osowa; X. Mazury, Giżycko; 3. Augustów Primeval Forest; 4. Pomerania, Szczecinek; 5. Greater Poland, Porażyn; 6. Wdzydze Landscape par, Kościerzyna; 7. Tuchola Pinewoods, Osiek; 8. Kujawy region, Toruń forests; 9. Warmia, Morąg; 10. Warmia, Olsztyn; 11. Warmia, Puchałowo; 12. Mazury, Piska Forest; 13. Sudety Mts., Kłodzka Dale; X. Mazury, Giżycko; Y. Tatra Mountains, Chochołowska Valley.

The *Boletus edulis* samples were obtained across the forested/woodland areas in Poland, ranging from the Baltic Sea south coast in the north to the Sudety and Tatra Mountains in the south (Figure 1). The composite samples of the whole fruiting bodies from 15 sampling sites altogether, caps from 5 sites, and stipes from 4 sites were examined and consisted of the young to mature (with white and yellow hymenophore, respectively) individuals, all being considered edible (Table 1).

An analytical scheme for the determination of trace elements was presented in detail in earlier studies [54,55,56]. In brief, dried mushrooms were ground to a fine powder and mixed. Subsamples of around 200 mg in duplicates were mixed with a 3 mL solution of ultrapure nitric acid (65%) and 1 mL of ultrapure hydrofluoric acid in polytetrafluoroethylene (PTFE) tubes. The tubes were screw-tightened in stainless-steel jackets and oven heated at 150 °C for 78 h. To remove the excess of hydrofluoric acid, the digests were concentrated at 110 °C to dryness, and the solid residue was dissolved in 1 mL of nitric acid and transferred up to a sample tube to give a final volume of 50 mL. Rhodium (Rh) (10–20 µg L^−1^) was used as an internal standard. All elements in the digested mushroom samples and certified reference materials were measured on the Quadruple ICP-MS analysis (Finnigan MAT ELEMENT, Thermo Fisher Scientific, USA) in triplicate, and the relative standard deviation values were always within 5% [54,57]. The certified reference materials utilized to check accuracy included the citrus leaves (GBW 10020) and soil (GBW 07405) [58]. The statistical data and graphical presentation of the results of two-dimensional multiple scatter plots, and the relationships between the variables determined, were obtained using Statistica software, version 13.1 (Statsoft Polska, Kraków, Poland) [56].

## 3. Results and Discussion

Each pooled sample of mushrooms (whole, caps or stipes) showed detectable quantities of Ag, As, Ba, Be, Bi, Cd, Co, Cu, Cs, Ga, Ge, Hf, Hg, In, Li, Mo, Nb, Ni, Pb, Rb, Sb, Sn, Sr, Ta, Th, Ti, Tl, U, V, W, Zn, or Zr. They contained also all 14 rare earth elements (La, Ce, Pr, Nd, Sm, Eu, Gd, Tb, Dy, Ho, Er, Tm, Yb, and Lu), which are presented and discussed here as sums (ƩREEs) (Table 1). For the purpose of normalization and for better readability, the original results on concentration levels and these cited from the literature were rounded up to two significant figures.

The composite samples of the whole fruiting bodies were characterized by a low coefficient of variation (<20%) for As, Cu, Ge, Hg, Ni, V, and Zn between the sites. Amongst the elements determined, Se, Cu, and Zn can be considered as the major trace micronutrients for consumers and for the physiological requirements of the fungus itself [7]. Due to a high biodiversity of wild species, there were around 2000 mushrooms classified as “edible” worldwide, and discussion of data obtained was focused and reduced to what is known on mineral nutrients of *B. edulis* or similar species reported from other studies.

### 3.1. Essential (Zn, Cu, Mo, and Co)

**Figure 2:**
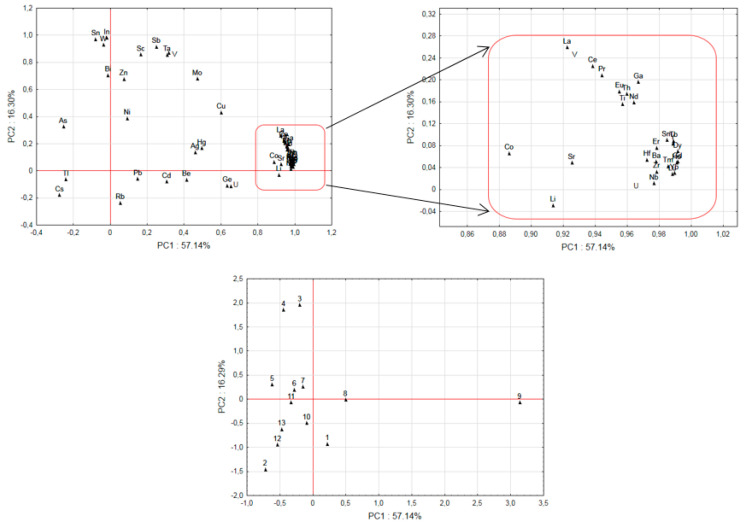
Plot of loadings based on the concentration of elements in fruiting bodies and projection of the element concentration levels in fruiting bodies collected in different sites set on the PC1 and PC2-plane, respectively (1. Coastal Landscape Park; 2. TLP, Osowa; 3. Augustów Primeval Forest; 4. Pomerania, Szczecinek; 5. Greater Poland, Porażyn; 6. WLP, Kościerzyna; 7. Tuchola Pinewoods, Osiek; 8. Toruń forests; 9. Morąg; 10. Olsztyn; 11., Puchałowo; 12. Piska Forest; 13. Kłodzka Dale).

**Figure 3:**
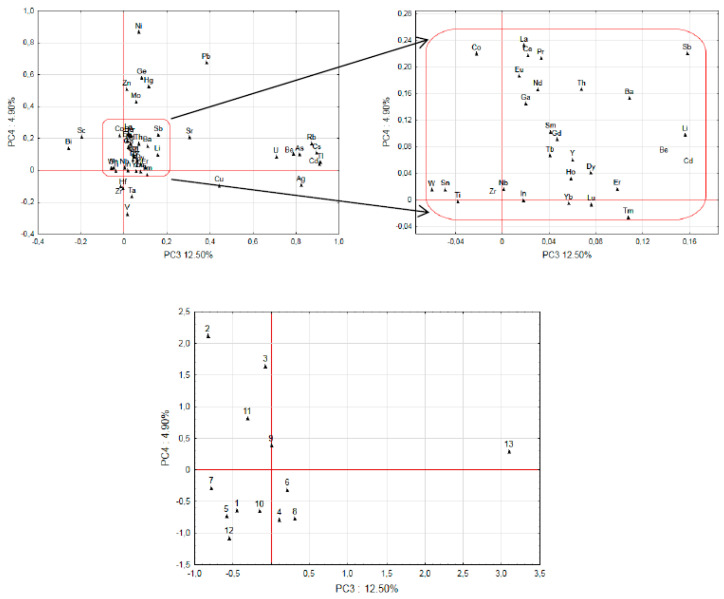
Plot of loadings based on the concentration of elements in fruiting bodies and projection of the element concentration levels in fruiting bodies collected in different sites set on the PC3 and PC4-plane, respectively (site id as in Figure 2).

The median values of the Zn, Cu, Mo, and Co concentrations in *B. edulis* were at the levels of 87, 25, 0.091, and 0.08 mg kg^−1^ dw, respectively (Table 1). The concentration levels of Zn and Cu showed relatively small variations (Table 1, Figure 2 and Figure 3), which could be related to the physiological and nutritional amounts required for this species to function. The distribution of elements between the caps and stipes in the fruiting bodies (Q_C/S_; cap to stipe concentration quotient) was in the favor of caps for Zn, Cu, and Mo (Q_C/S_ from 1.8 to 2.0), while vice versa was found for Co (Q_C/S_ at 0.7).

The physiological requirements of macrofungi with respect to essential elements under typical forest topsoil/environmental conditions for different species could be reflected through their concentration levels sequestered in fruiting bodies. A small variation in Zn and Cu concentration levels in mushrooms from spatially distant sites suggests typical enrichment and content in soil substrata, where mycelium is found. It is worth noticing that mycelium of *B. edulis* is found in deeper topsoil compared to many other forest mushrooms, and this has consequences for, e.g., temporal trends observed in contamination with radiocaesium [51].

Zn and Cu accumulated in fruiting bodies of *B. edulis* can be of natural occurrence in soil substrata arising from parent bedrock or can be due to soil pollution with these elements. Nikkarinen et al. (2004) found higher accumulation both of Zn and Cu in *B. edulis* grown in a region with a sulphide ore belt contained within the parent soil bedrock (92 (46–140) mg kg^−1^ dw (n = 8) for Zn and 42 (24–94) mg kg^−1^ dw for Cu (n = 3)), when compared to a region with granite as a parent soil bedrock (64 (51–81) mg kg^−1^ dw and 20 (15–27) mg kg^−1^ dw, respectively) [33]. A more pronounced impact on the accumulation of Zn and Cu was observed because of soil pollution due to fumes released from a zinc smelter and a copper smelter. In a study by Collin-Hansen et al. (2005) [58], Zn in caps of *B. edulis* from a polluted forest jumped to a level of 450 ± 190 mg kg^−1^ dw (zinc smelter) and 250 ± 71 mg kg^−1^ dw (copper smelter) and Cu up to 78 ± 31 mg kg^−1^ dw (zinc smelter) and 290 ± 260 mg kg^−1^ dw (copper smelter). Typical concentrations of Zn and Cu in caps of *B. edulis* from reference (unpolluted) sites for both smelters mentioned were in the range from 120 ± 15 to 150 ± 23 mg kg^−1^ dw and from 20 ± 3 to 57 ± 12 mg kg^−1^ dw, respectively [59]. Typical concentrations of Zn reported for *B. edulis* from other sites and considered as unpolluted in Europe were: 170 ± 34 mg kg^−1^ dw (n = 3), 110 (100–120) mg kg^−1^ dw, from 84 ± 3 to 130 ± 4 mg kg^−1^ dw, 70 mg kg^−1^ dw (mean in whole mushrooms), and 120–120 mg kg^−1^ dw (medians for caps) and 58–60 mg kg^−1^ dw (medians for stipes) [26,28,37,40,46].

For Cu, the concentration levels determined in *B. edulis* from Poland (Table 1) showed typical values as determined in specimens from an unpolluted sites elsewhere in Europe, which (whole mushrooms), depending on the authors, were: 19 ± 5 (n = 3) mg kg^−1^ dw, from 21 ± 3 to 59 ± 5 mg kg^−1^ dw, 23 (22–23) mg kg^−1^ dw and 15 mg kg^−1^ dw and from 24 to 42 mg kg^−1^ dw (medians for caps) and 11-17 mg kg^−1^ dw (medians for stipes) [26,28,37,40,46].

Mo (molybdenum) occurs as a micronutrient in *B. edulis*, and caps were richer than stipes (the medians were 0.095 and 0.054 mg kg^−1^ dw, respectively; Table 1). This element in the whole fruiting bodies was in the range from 0.054 to 0.13 mg kg^−1^ dw, in agreement with a sole observation available from literature for this species collected at unpolluted regions of Finland, where it occurred in the range from 0.06 to 0.13 mg kg^−1^ dw [33].

Co (cobalt), like Mo, is a micronutrient in *B. edulis*. Except for the sites Giżycko and Morąg in the Warmia-Masuria region and the Tatra Mts site, where mushrooms showed Co in the range from 0.37 and 0.85 mg kg^−1^ dw, which is higher when compared to the remaining localities where up to 0.13 mg kg^−1^ dw was found (Table 1, Figure 2 and Figure 3). Available data on Co in *B. edulis* worldwide are limited. The results obtained seem comparable as reported by some authors in spite of a high geospatial variability across the sites considered and also given the variation in parent soil bedrock composition, i.e., the concentration levels were: 0.050 ± 0.005 to 0.058 ± 0.008 mg kg^−1^ dw, 0.05 (0.02–0.10) mg kg^−1^ dw, 0.08 (0.05–0.15), and 0.08 (0.07–0.09) mg kg^−1^ dw [26,28,33]. In mushrooms from a site in Serbia, the mean value was higher, i.e., 0.72 mg kg^−1^ dw [46].

### 3.2. Toxic Elements (Ag, As, Cd, Hg, Pb, Sb, Tl, and U)

Ag, when compared with other toxic elements studied, was found at relatively higher concentrations (Table 1). The median concentration of Ag in the whole mushrooms was 2.9 mg kg^−1^ dw, and, for other toxic elements (mg kg^−1^ dw), the medians were: 1.8 (Cd), 1.4 (Hg), 0.96 (Pb), 0.47 (As), 0.095 (Tl), 0.024 (Sb), and 0.0065 (U) (Table 1). Depending on the site of collection, the maximum concentration for Ag, As, Cd, Hg, Pb, Sb, Tl, or U in mushroom was: Ag at 6.5 mg kg^−1^ dw, Cd at 4.8 mg kg^−1^ dw, As at 0.88 mg kg^−1^ dw, and Tl at 0.28 mg kg^−1^ dw for specimens collected from the region of the Sudety Mts; Hg at 2.2 mg kg^−1^ dw from the Giżycko site; Pb at 1.6 and 1.5 mg kg^−1^ dw and U at 0.028 and 0.028 mg kg^−1^ dw, respectively, at the Sudety Mts and Morąg sites; and Sb was at 0.057 mg kg^−1^ dw in those from the Augustów Primeval Forest. In the case of uranium, the caps of specimens collected from the Tatra Mts showed an elevated concentration reaching a maximum of 0.038 mg kg^−1^ dw (stipes were not available for analysis) (Table 1).

*B. edulis* can efficiently bioconcentrate Ag in fruiting bodies [35]. Relatively high concentrations of Ag were found in specimens grown at sites with a parent soil bedrock composed of the sulphide ore belt (shales), which accumulated 14 ± 0 mg kg^−1^ dw and 16 mg kg^−1^ dw, and less was found in specimens gathered from regions with other types of a parent soil bedrock (paelozolic graywacke or granite), where levels were substantially lower, i.e., 1.9 ± 0.1 mg kg^−1^ dw and 4.3 mg kg^−1^ dw [26,33]. In previous studies in Poland, Ag in the whole *B. edulis* was present in the range from 0.16 to 2.6 mg kg^−1^ dw, when using dry ashing to decompose the organic matrix and flame atomic absorption spectroscopic determination [40], and, in another study, the caps showed 22 ± 6 mg kg^−1^ dw (no data for stipes), after nitric acid oxidation of a matrix and ICP-sector field spectrometric determination [29].

Ag is often associated with copper ores [60,61]; smelting and refining of the copper ores is a known source of environmental pollution for Cd and Hg resulting in well-elevated concentration levels of both elements in *B. edulis* within the proximity of a smelter [59], but, on the link has not been established for Ag. Metallic silver in dentures is known to be carcinogenic, and nano-Ag is widely used nowadays in nanotechnology due to its anti-bacterial properties [62]. Silver ions are highly toxic and are reactive (chalcophile). They can react immediately with any sulfhydryl groups (–SH) of tissues and denature peptides/proteins. Use of the nano-Ag and its release from waste-water-treatment plants creates toxicological problems in the aquatic environment [63]. The nano-Ag is adsorbed by sewage sludge [64], and crops can be exposed directly to Ag nanoparticles and their transformed particles from contaminated sewage sludge if it is applied to agricultural or forest soils as a fertilizer.

The composite samples of caps showed Ag, Cd, and Hg at higher concentrations (*p* < 0.05) than stipes, while Tl was higher in stipes than caps (*p* < 0.05). The distribution of Ag, Cd, Hg, Sb, and Tl in fruiting bodies followed a pattern noticed earlier in specimens from some other places as determined by other authors, while only a few data were reported previously for As, Pb, and U [31,34,35,36,42].

The medians of Cd concentrations determined in the fruiting bodies, caps, and stipes in the present study were 1.8, 3.0, and 0.9 mg kg^−1^ dw, respectively, and specimens collected from the montane regions showed Cd in caps at higher levels than elsewhere (Table 1) (3.6 mg kg^−1^ dw from the Tatra Mts site and 6.9 mg kg^−1^ dw from the Sudety Mts site). As can be concluded from the studies by Nikkarinen et al. (2004) and Řanda and Kučera (2004), the parent soil bedrock made of the sulphide ore belt or Precambrian shales can have an impact on the occurrence of Cd in *B. edulis*, and concentrations were 3.4 mg kg^−1^ dw (belt) and 5.1 ± 0.6 mg kg^−1^ dw (shales), if related to bedrock of other type with 1.4 mg kg^−1^ dw (granitic) and 2.1 ± 0.3 mg kg^−1^ dw (paelozolic graywacke) [26,33]. Nevertheless, the effect of the occurrence of Cd in topsoil (humus layer) seemed to be more evident and sound for *B. edulis* than its occurrence in subsoil (minerogenic parent soil) [33].

Cd, like Ag, is also efficiently bioconcentrated by *B. edulis* in fruiting bodies (bioconcentration factor for caps in the range from 12 ± 3 to 30 ± 6), and mushroom shows some potential for bio-indication of this element at a weakly polluted soils [35]. A study by Collin-Hansen et al. [59] showed links between sites substantially polluted with Cd (also Zn and Cu) due to the proximity of a metal/ore smelter. *B. edulis* grown in soils polluted with Cd from the zinc or copper smelters were highly contaminated with this element, e.g., in caps, it was 120 ± 65 mg kg^−1^ dw (zinc smelter) and 19 ± 10 mg kg^−1^ dw (copper smelter) [59]. *B. edulis* (caps) was collected from the sites affected by an abandoned lead smelter in Slovenia (Meža Valley) or a thermal power plant (Šalek Valley) and showed medians of Cd at 27 mg kg^−1^ dw and 7.5 mg kg^−1^ dw, and at the reference site, it was 1.7 mg kg^−1^ dw [43].

The medians of Pb concentrations in the whole fruiting bodies, caps, and stipes were 0.96, 0.72, and 0.66 mg kg^−1^ dw, respectively, and the maximum value in whole mushrooms was 1.8 mg kg^−1^ dw. Since the spread of industrialization, the element Pb has persisted as a notorious contaminant in topsoils at regions around the world. The contamination of *B. edulis* collected at the Osowa and Morąg sites, with Pb in the range from 1.8 to 1.5 mg kg^−1^ dw, could be due to anthropogenic pollution (traffic, leaded gasoline, and spent ammunition, respectively), while in the case of mushrooms from the Sudety and Tatra Mts sites, it could be associated with parent soil bedrock composition (Table 1). In general, concentrations of Pb noted in this study were close to the mean value of 0.56 mg kg^−1^ dw (range 0.11–1.6 mg kg^−1^ dw) as determined in the reference *B. edulis* samples collected from an unpolluted site in Slovenia, while in the vicinity of a Pb smelter, concentrations were in the range from 0.76 to 12 mg kg^−1^ dw (median 4.8 mg kg^−1^ dw) [43].

The medians of Hg concentrations in the whole fruiting bodies, caps, and stipes were 1.4, 1.4, and 1.1 mg kg^−1^ dw, respectively (Table 1). Caps showed Hg in the range from 1.3 to 2.9 mg kg^−1^ dw and stipes from 0.57 to 1.5 mg kg^−1^ dw (some asymmetry in the number of the pools between the sample sets).

Total mercury (THg) and methylmercury (MeHg) were recently studied in a set of crude and braised *B. edulis* during various developmental stages and in several sets of crude specimens [13,65]. The THg in crude fruiting bodies of a different developmental stage was in concentration 2.4 ± 0.3 mg kg^−1^ dw (0.24 ± 0.03 mg kg^−1^ wet weight, ww) and when braised was less on dry weight. i.e., 1.4 ± 0.4 mg kg^−1^ dw but more on wet weight, i.e., 0.36 ± 0.09 mg kg^−1^ ww. In parallel, MeHg content in crude mushrooms was 0.042 ± 0.010 mg kg^−1^ dw (0.0042 ± 0.0010 mg kg^−1^ ww) and in braised was 0.021 ± 0.008 mg kg^−1^ dw (0.0053 ± 0.0022 mg kg^−1^ ww), and the contribution of MeHg in THg was similar in both, i.e., 1.0 to 3.3% in crude and 0.8 to 1.7% in braised [13]. The contribution of MeHg to THg varied widely, i.e., from 0.50 to 4.1% in sets of crude *B. edulis* reported in other studies, while their concentrations were in the rages from 0.35 to 3.1 mg kg^−1^ dw and from 0.011 to 0.077 mg kg^−1^ dw [65]. Selenium is known due to its occurrence and function as an essential component of selenoenzymes and also as a major target for absorbed, toxic MeHg. The concentration of Se in mushrooms is important for potential protection from toxic effects of MeHg and inorganic Hg.

The medians of Sb concentrations considered as most important in this case in the whole fruiting bodies, caps, and stipes were 0.024, 0.026, and 0.017 mg kg^−1^ dw, respectively (Table 1). The obtained results show a low concentration of Sb; while only a few data for this species are available from the literature, the amounts reported were comparable, e.g., from below 0.02 and 0.06 ± 0.01 mg kg^−1^ dw [26]. *B. edulis* collected at highly polluted places in the vicinity of the abandoned mines in the Čučma, Medzibrod and Dúbrava regions deposits, with ores there such as stibnite (Sb_2_S_3_), berthierite (FeSb_2_S_4_), and jamesonite (Pb_4_FeSb_6_S_14_), zinkenite (Pb_9_Sb_22_S_42_), tetrahedrite [(Cu,Fe)_12_Sb_4_S_13_], and ores of other elements (As, Fe, Zn), accumulated Sb in the range from 2.0 to 3.2 mg kg^−1^ dw. Higher concentrations from 32 to 360 mg kg^−1^ dw (rounded) were found in three *Suillus* species of mushroom [66].

The medians of Tl concentrations in the whole fruiting bodies, caps, and stipes were 0.095, 0.052, and 0.14, mg kg^−1^ dw, respectively, and typically Tl was higher in stipes than caps (Table 1). The place of collection, and possibly the occurrence of Tl in the parent bedrock, can impact its accumulation in *B. edulis*, as it could be observed for specimens from the Sudety Mts, which showed statistically higher contamination (the medians were 0.16, 0.40, and 0.28 mg kg^−1^ dw in the whole mushrooms, caps, and stipes, respectively) (Table 1, Figure 2). The results in this study agree with data reported so far on Tl in *B. edulis* sampled elsewhere, i.e., it was 0.027 ± 0.012 mg kg^−1^ dw (caps) and from below 0.3 mg kg^−1^ dw (whole mushrooms) [26,29].

U is a natural but radiotoxic element (^234^U, ^235^U, ^236^U, and ^238^U isotopes are emitters of highly toxic alpha-particles, consisting of a 4He nucleus with two protons and two neutrons), which is ubiquitous in food webs including wild mushrooms, while the type of emission varies with the isotope [49]. The Quadruple ICP-MS analysis allows for rapid and accurate measurement of the total U in a digested sample. Until now, no specific accumulator of U has been found among mushrooms. In a literature search, the typical levels of U in *B. edulis* were: 9.7 ± 1.2 (8.2–11) mg kg^−1^ dw in caps, and from 2.7 to 6.9 mg kg^−1^ dw and from 0.012 ± 0.003 to < 0.040 mg kg^−1^ dw in the whole mushrooms [26,29,67]. This element, as reviewed, has values of bioconcentration factor (BCF) in the range from 0.005 to 0.19, and so is classified as bioexcluded [49]. The sampling site seems to be major determinant of the quantity of U accumulated in mushrooms. *B. edulis* from the Sudety Mountains and Morąg sites had relatively higher concentrations of U, and this can be explained by the geology of the site of collection. The Sudety Mts is a region of U ores [68], resources largely exploited in Poland by the Soviet Union after the period following the end of the World War II. In the Morąg region, military use of the site in the past can be considered as a source of topsoil contamination with U. As reviewed recently, studies of the European mushrooms showed that activity concentration of the U radioisotopes exhibit some spatial differences that can be related to its background occurrence in parent bedrock for soils and anthropogenic sources, while levels of this little bioconcentrated element by *B. edulis* and other species were considered safe for human [49].

In the European Union (EU), there is no regulation on allowable concentration levels of Ag, As, Cd, Hg, Pb, Sb, and Tl in wild mushrooms, while the occurrence of radioactive uranium species undergoes risk-assessment analysis as the alpha-particle emitters [49]. In the EU, there is regulation on allowable levels of Cd and Pb for three species of a cultivated mushrooms at the fresh state: *Agaricus bisporus*, *Pleurotus ostreatus*, and *Lentinula edodes*, and this regulation is sometimes used as a basis for safe levels of elements in other mushrooms [61]. Toxic Cd and Pb were in *B. edulis* at concentration levels below the limits set by the European Union regulations for cultivable species, while Ag, As, Hg, Sb, Tl, and U, which are not regulated in mushrooms, were at relatively low or typical concentrations as usually found in mushrooms from unpolluted regions.

### 3.3. Monovalent Elements (Cs, Li, Rb)

The median values of the Cs, Li, and Rb concentrations in the whole fruiting bodies were 1.8, 0.075, and 190 mg kg^−1^ dw, respectively (Table 1). All three of these elements are not considered as essential for fungi or human nutrition, while debate on the essential nature of Li is ongoing [69,70,71,72,73]. Typical occurrences of Li in different foods including many mushrooms and intakes are low [33,69,70,71,72,73,74,75]; however, Li is a widely used metal in industrial and domestic appliances. It can be considered as a potential food contaminant due to its prevalence. In the case of Cs and mushrooms, the major problem seems to be due to ongoing contamination with radioactive caesium (^137^Cs) [50,76,77,78], rather than with the stable isotope Cs (^133^Cs) [79,80].

Rb, the next major monovalent cation after potassium (K) in mushrooms, occurs typically at concentrations of around 350 mg kg^−1^ dw in caps of *B. edulis* [2]. After K and Rb is sodium Na, which tends to be at greater concentration in the stipes (340 mg kg^−1^ dw, medians) than the caps (100–200 mg kg^−1^ dw) of this mushroom [34]. The stipes of *B. edulis*, based on a relatively large set of samples, were around 1.5 times lower in Rb than caps (Table 1). This observation agrees with an earlier one that was based on a smaller set of mushroom samples and a different analytical methodology [34]. Based on the results from this study and from published data, it can be concluded that Rb in the morphological parts of *B. edulis* follows the fate of K, which occurred in a similar ratio of the cap to stipe concentration quotient (Q_C/S_) and regardless of the absolute concentration values—as observed in other studies [34,36,37].

### 3.4. The Elements Ba, Sr, Ni and V

The elements Ba, Sr, Ni, and V occurred in fruiting bodies at median concentrations of 1.5, 0.48, 1.5, and 1.6 mg kg^−1^ dw, respectively (Table 1). However, in the case of Ba and Sr, differences between the sites were substantial (coefficient of variation was greater than 100%). Ba and Sr are elements associated with each other in nature and both also strongly correlate with calcium (Ca) in mushrooms including *B. edulis* [34]. The Ba median concentrations in the pools of caps and stipes were 1.2 and 2.1 mg kg^−1^ dw; the Sr concentrations were 0.37 and 0.54 mg kg^−1^ dw; and the Ni concentrations were 1.6 and 1.3 mg kg^−1^ dw, respectively (Table 1). The Q_C/S_ values of both Ba and Sr were in the favor of stipes (Q_C/S_ 0.57 and 0.68, respectively), and for Ni, the value was 1.2. Differences in their distribution were statistically significant (*p* < 0.05). All these three elements were considered minor constituents in *B. edulis*. Some literature data shows that Ba in the occurring specimens collected from the sites with different parent soil bedrock can be found with concentrations of 0.27 (0.18–0.41) mg kg^−1^ dw (sulphide belt) and 0.16 (0.10–0.19) mg kg^−1^ dw (granite) [33] and in a collection grown on sandy soils concentrations ranged from 0.19 to 0.63 mg kg^−1^ dw (medians) in caps and from 0.48 to 0.51 mg kg^−1^ dw in stipes [46]. Similarly, Sr was in concentrations from 0.22 (0.15–0.30) mg kg^−1^ dw (sulphide belt) and 0.22 (0.09–0.33) mg kg^−1^ dw (granite) to 1.0 ± 0.2 mg kg^−1^ dw (Precambrian shales) and 2.1 mg kg^−1^ dw (unpolluted region in a rural Serbia), and in mushrooms from sandy soils, medians were in the range from 0.06 to 0.22 mg kg^−1^ dw in caps and from 0.20 to 0.26 mg kg^−1^ dw in stipes [26,33,37,46].

The median values of Ni in the whole fruiting bodies, caps, and stipes were 1.6, 1.4, and 1.5 mg kg^−1^ dw, respectively (Table 1). *B. edulis* collected in Finland contained Ni at concentrations of 0.91 mg kg^−1^ dw (n = 1) [28] and was 1.8 mg kg^−1^ dw (range 1.2–2.5 mg kg^−1^ dw) in specimens grown on a sulphide ore belt and 1.6 mg kg^−1^ dw (range 1.3–1.8 mg kg^−1^ dw) in specimens collected from an area with granite bedrock [33]. Additionally, in rural Serbia, Ni was found in *B. edulis* at typical levels, i.e., 1.2 mg kg^−1^ dw [46]. Řanda and Kučera in specimens taken from the Paelozolic graywacke and Precambrian shale bedrocks determined Ni at below 13 and 9.0 ± 2.0 mg kg^−1^ dw [33]. Ni is not essential to humans [73], and its presence at low concentrations in mushrooms, as defined in studied *B. edulis*, is common [32].

The minimum and maximum values of vanadium in the whole mushrooms at the sites were 0.77 and 2.2 mg kg^−1^ dw (Table 1), and typical levels were observed so far in *B. edulis* and some other species. An exception was found for two *Amanita* spp., i.e., *A. muscaria* and *A. velatipes*, for which the element V is essential and occurs at concentrations typically exceeding 100 mg kg^−1^ dw [81,82]. The distribution of V between the caps and stipes of *B. edulis* was similar (Q_C/S_ = 1).

### 3.5. Elements Rarely Studied in B. edulis: Be, Bi, Ga, Ge, Hf, In, Nb, Sn, Ta, Th, Ti, W, Zr, ƩREEs

The elements Be, Bi, Ga, Ge, Hf, In, Nb, Ta, Th, and W, and also ƩREEs, were only found at low levels in *B. edulis*, i.e., at the concentrations from below 0.1 to below 0.01 mg kg^−1^ dw, and for Ʃ14 REEs, the median was 0.31 mg kg^−1^ dw (Table 1). The credibility of a measurements is a key question at very low concentrations in mushrooms [67,83] and is an important consideration when comparing and interpreting results. Elements such as Be, Bi, Ga, Ge, Hf, In, Nb, Sn, Ta, Th, Ti, W, Zr, and ƩREEs when found in mushrooms, and other foods at typical levels, are not considered as having any consequences for health in terms of either benefits or harmful effects [29,54,55,73]. Some of them, i.e., Be, Ga, In, Ta, and Ti, were nearly equally distributed between the stipes and caps (Q_C/S_ from 0.93 to 1.2), and Hf, Nb, Th, and Zr were higher in stipes (Q_C/S_ from 0.47 to 0.67), while Bi, Ge, and W were higher in caps (Q_C/S_ from 1.4 to 1.6).

Sn and Zr occurred at concentrations below 1 mg kg^−1^ dw, and only Ti was present at much higher concentrations with a median value in the whole mushrooms at 18 mg kg^−1^ dw with a maximum value of 57 mg kg^−1^ dw (Table 1). The median concentration of Zr in the whole mushrooms was 0.70 mg kg^−1^ dw, while the content differed for mushrooms from different locations and was in the range from 0.11 mg kg^−1^ dw in the Osowa site up to 5.0 mg kg^−1^ dw in the Morąg site. Curiously, mushrooms from Řanda and Kučera were found to contain Zr in a few specimens of *B. edulis* (Precambrian shales) at concentrations of 5.6 ± 0.7 mg kg^−1^ dw [26]. The pools of caps of the saprotrophic *Macrolepiota procera* collected across of Poland showed Zr at 1.1 ± 0.6 mg kg^−1^ dw [54].

Beryllium (Be) was determined in the whole mushrooms, caps, and stipes and was found to have median concentrations of 0.023, 0.026, and 0.021 mg kg^−1^ dw, respectively (Table 1). Previous data published on Be in *B. edulis*, in a report from Finland, showed it was found in the range from 0.01 to 0.02 mg kg^−1^ dw [33], and no other results could be identified from the available literature. The Be in *Morchella* mushrooms from Sicily was not found above a limit of quantification at 0.03 mg kg^−1^ dw [79]. No previous data on Ga, Ge, and Sn in *B. edulis* could be identified in the available literature.

Bi was found in the present study to be in *B. edulis* at concentrations similar to those reported previously for this species using the sector-field mass spectrometry (ICP-HRMS), i.e., at 0.0012 ± 0.0006 mg kg^−1^ dw [29]. Those results are of two orders of magnitude lower than noted in this species from Serbia, which showed Bi at 0.44 mg kg^−1^ dw [46]. The result for hafnium in the whole mushrooms was at 0.015 mg kg^−1^ dw, which agrees with reported data for two specimens of *B. edulis* when determined by instrumental neutron activation analysis (INAA) at the short-term mode, which showed from below 0.030 mg kg^−1^ dw to below 0.020 mg kg^−1^ dw [26]. Another example of the ultra-trace element in *B. edulis* is indium (In), which occurred at 0.008 mg kg^−1^ dw (caps with 0.0052 mg kg^−1^ dw; Table 1). In another study, indium was in caps at 0.0032 ± 0.0012 mg kg^−1^ dw) [29]. Niobium was determined at 0.022 mg kg^−1^ dw (Table 1), which differs by one order of magnitude from a result of 0.0031 ± 0.0003 mg kg^−1^ dw as determined in a single mushroom by instrumental photon activation analysis (IPAA) [26].

The element tantalum (Ta) occurred in the whole *B. edulis* and in caps and stipes alone in the range from 0.012 to 0.020 mg kg^−1^ dw (Table 1). Tantalum was below 0.010 kg^−1^ dw in a few individuals examined by INAA [26]. Thorium (Th) was at median concentration of 0.010 mg kg^−1^ dw, with the exception of the Morąg site where the concentration was 0.070 mg kg^−1^ dw (a place associated with a legacy pollution problem), whilst the range for other sites was much lower, i.e., from 0.0022 to 0.026 mg kg^−1^ dw (Table 1). Previously, Th was reported in caps of *B. edulis* at 0.0040 ± 0.0023 mg kg^−1^ dw (ICP-sector field mass spectrometry), and in the whole mushrooms was below 0.020 mg kg^−1^ dw (INAA) and from 0.00067 to 0.00157 mg kg^−1^ dw (ICP-MS) [26,29,67].

### 3.6. Principal Component Analysis

The PCA with the correlation matrix was performed to investigate possible relationship between the elements contained in *B. edulis* and to differentiate mushrooms with respect to 13 collection sites (Table 1; Figure 1, Figure 2 and Figure 3). Four main components (*p* < 0.05) could be selected (all factor coordinates with positive charge) that were related to 88% of total variance and with PC1 (57%) determined by Ba, Co, Ga, Li, Nb, Sr, Th, Ti, Y, Zr, and ƩREEs; PC2 (16%) by Bi, In, Sc, Sb, Sn, Ta, V, and W; PC3 (12%) by Ag, As, Be, Cd, Cs, Rb, Tl, and U; and PC4 (4.9%) by Ni.

The Morąg site (id 9) was well separated from the others, due to the loadings covered under PC1 (Figure 2) and the Sudety Mts site (id 13) covered under PC3 (Figure 3). The sites at the Augustów Primeval Forest (id 3) and Szczecinek (id 4) were separated from others due to influence from the elements from the PC2 (Figure 2), while the Osowa site (id 2) was separated from the PC 4; the Augustów Primeval Forest site (id 3) was separated because of Ni and in part also because of Pb (PC 4). Mushrooms from the site Osowa (id 2) that were relatively rich in Pb (Pb from leaded gasoline has been identified as major source of this element to saprotrophic *M. procera* [84]) were also low in ƩREEs as well as Bi, Sc, V, In, Sn, Sb, Ta, W, and Zn (Figure 3). On the other hand, for the elements not covered by PC1–PC4, there was no statistically significant difference (*p* > 0.05) for the sites. When taking into account the morphological parts of a fruit body, and recognizing that there were only a limited number of pooled samples, the elements Ag, Cd, Cu, Cs, Ga, Ge, Hg, Mo, Ni, Rb, Sb, Ti, and Zn were at higher concentrations in caps than stipes, while Ba, Co, Hf, Sr, Tl, and Zr were higher in stipes than caps (*p* < 0.05).

Mushrooms, both cultivated and edible wild, have to be cooked to avoid food poisoning. Recipes are abundant, and some species are considered inedible in some places yet edible elsewhere. This could be associated with knowledge or local secrets of preparation—see in Finland [85], where human longevity is relatively high as is consumption of wild edible mushrooms. Mushrooms for human consumption have to be culinary processed, and nowadays only relatively few species are consumed in the raw state, e.g., *Tricholoma matsutake*. Culinary processing has an impact on the composition and concentration of chemical compounds and their intake from a mushroom product or meal, while this point is extremely rarely considered during, e.g., “exposure and risk assessment” or “nutritional elements intake” assessments of the elements from mushrooms.

## 4. Conclusions

The study provided base data on the trace elements in *B. edulis*. Analysis using a validated procedure with measurement by quadrupole ICP-MS followed by a PCA statistical approach enabled characterization of mushroom collected from across regions of Poland with respect to natural occurrence or anthropogenic contamination of mushrooms with a range of trace elements (Ag, As, Ba, Bi, Cd, Co, Cu, Cs, Hg, Ni, Pb, Rb, Sb, Sr, Tl, U, V, W, and Zn). This included elements where reported data were lacking such as Be, Hf, In, Li, Mo, Nb, Sn, Ta, Th, Ti, and Zr, as well as ƩREEs. The concentrations measured displayed a somewhat scattered picture due to the impact of anthropogenic factors at 2–3 collection sites out of a total 15 but also due to the impact from geological (natural) factors. These findings may have a bearing on the suitability of *B. edulis* mushrooms for human consumption as a result of the presence of some of these metallic elements. However, the concentrations of the most notoriously toxic metallic elements in *B. edulis* were considered harmless. Mushrooms for human consumption have to be carefully prepared, and a banal question is that any consideration on their chemical composition and suitability, both for wild edible species as well as cultivated ones, as a raw food material need to be verified.

## Figures and Tables

**Table 1:** Elements in fruiting bodies of *B. edulis* (mg kg^−1^ dw).

Place (Number of Specimens and Morphological Part) *	**Ag**	**As**	**Ba**	**Be**	**Bi**	**Cd**	**Co**	**Cu**	**Cs**	**Ga**	**Ge**	**Hf**	**Hg**	**In**	**Li**		**Mo**
(1) Coastal Landscape Park (15; c)	3.1	0.58	1.2	0.026	0.0063	2.0	0.044	30	1.2	0.074	0.080	0.024	1.4	0.0016	0.14		0.082
(1) Coastal Landscape Park (15; s)	1.4	0.44	3.0	0.021	0.0086	0.82	0.028	26	2.8	0.065	0.057	0.059	1.3	0.0014	0.036		0.054
(1) Coastal Landscape Park (15; w)	2.2	0.51	2.1	0.023	0.0074	1.4	0.036	28	1.5	0.070	0.068	0.041	1.3	0.0015	0.088		0.068
(2) TLP, Osowa (10; w)	1.1	0.43	1.1	0.016	0.0013	1.2	0.055	20	2.3	0.050	0.097	0.0022	1.6	0.0011	0.033		0.068
(X) Mazury, Giżycko (15; c)	4.8	0.48	1.4	0.031	0.0072	2.6	0.56	27	2.0	0.073	0,093	0.0073	2.9	0.0072	0.060		0.13
(X) Mazury, Giżycko (15; s)	1.7	0.43	1.9	0.022	0.0038	0.98	0.71	12	0.95	0.055	0.057	0.017	1.5	0.0069	0.11		0.069
(X) Mazury, Giżycko (15; w)	3.2	0.45	1.6	0.026	0.0055	1.8	0.63	19	1.5	0.064	0.075	0.012	2.2	0.0070	0.085		0.10
(3) Augustów Primeval Forest (16; w)	3.1	0.67	2.7	0.024	0.026	2.2	0.13	25	1.7	0.089	0.079	0.015	1.8	0.021	0.081		0.12
(4) Pomerania, Szczecinek (22; w)	3.4	0.65	1.2	0.020	0.013	1.5	0.11	29	2.2	0.072	0.071	0.026	1.1	0.020	0.043		0.092
(5) Greater Poland, Porażyn (13; w)	2.8	0.55	0.64	0.011	0.017	1.4	0.021	21	1.3	0.046	0.056	0.010	1.5	0.012	0.014		0.063
(6) WLP, Kościerzyna (45; w)	3.4	0.47	1.5	0.033	0.0046	2.0	0.11	25	2.0	0.066	0.080	0.016	1.7	0.012	0.14		0.078
(7) Tuchola Pinewoods, Osiek (15; w)	2.1	0.44	1.5	0.017	0.025	1.2	0.087	22	1.2	0.073	0.064	0.020	1.1	0.0090	0.15		0.084
(8) Kujawy region, Toruń forests (16; w)	3.2	0.51	3.3	0.029	0.0022	2.9	0.12	27	2.3	0.086	0.086	0.046	1.3	0.010	0.069		0.083
(9) Warmia, Morąg (30; w)	1.4	0.41	3.0	0.035	0.0084	0.82	0.85	31	1.1	0.18	0.11	0.11	2.0	0.0014	0.49		0.054
(10) Warmia, Olsztyn (19; w)	2.4	0.43	1.6	0.021	0.0021	2.1	0.028	26	2.8	0.065	0.057	0.026	2.0	0.0056	0.036		0.082
(11) Warmia, Puchałowo (15; w)	1.5	0.48	1.3	0.027	0.0041	2.2	0.073	21	2.5	0.075	0.09	0.0047	1.4	0.010	0.026		0.094
(12) Mazury, Piska Forest (15; c)	4.3	0.39	0.44	0.010	0.0004	3.0	0.047	24	2.2	0.057	0.088	0.0011	1.3	0.0050	0.050		0.080
(12) Mazury, Piska Forest (15; s)	1.4	0.39	0.69	0.017	0.00044	0.82	0.079	11	1.2	0.028	0.046	0.0027	0.57	0.0053	0.040		0.038
(12) Mazury, Piska Forest (15; w)	2.8	0.39	0.56	0.013	0.00042	1.9	0.063	17	1.7	0.042	0.067	0.0019	0.93	0.0051	0.045		0.059
(13) Sudety Mts., Kłodzka Dale (15; c)	9.5	1.0	1.8	0.044	0.0009	6.9	0.070	34	7.2	0.065	0.088	0.0064	1.9	0.0052	0.19		0.095
(13) Sudety Mts., Kłodzka Dale (15; s)	3.5	0.76	2.4	0.046	0.0037	2.7	0.12	19	4.3	0.051	0.064	0.012	1.0	0.0058	0.095		0.053
(13) Sudety Mts., Kłodzka Dale (15; w)	6.5	0.88	2.2	0.042	0.0063	4.8	0.095	26	5.7	0.058	0.076	0.0092	1.4	0.0055	0.14		0.074
(Y) Tatra Mountains, Chochołowska Valley (12; c)	9.2	0.51	0.84	0.012	0.056	3.6	0.37	28	1.7	0.055	0.039	0.045	1.4	0.011	0.12		0.10
Mean caps (5 composites)	6.2	0.59	1.1	0.025	0.014	3.6	0.22	29	2.9	0.065	0.078	0.017	1.8	0.0060	0.11		0.097
SD	3.0	0.24	0.5	0.014	0.024	1.9	0.23	4	2.4	0.009	0.022	0.018	0.7	0.0030	0.06		0.020
Median	4.8	0.51	1.2	0.026	0.006	3.0	0.07	28	2.0	0.065	0.088	0.007	1.4	0.0052	0.12		0.095
Mean stipes (4 composites)	2.0	0.50	2.0	0.026	0.0041	1.3	0.23	17	2.3	0.050	0.056	0.023	1.1	0.0048	0.070		0.054
SD	0.9	0.15	0.8	0.011	0.0029	0.8	0.28	6	1.3	0.013	0.006	0.022	0.3	0.0021	0.032		0.011
Median	1.5	0.44	2.1	0.021	0.0037	0.9	0.10	15	2.0	0.053	0.052	0.014	1.1	0.0055	0.067		0.054
Mean whole (14 composites)	2.8	0.52	1.7	0.024	0.0088	2.0	0.17	24	2.1	0.074	0.077	0.024	1.5	0.009	0.10		0.080
SD	1.3	0.13	0.8	0.009	0.0084	1.0	0.25	4	1.1	0.033	0.015	0.028	0.4	0.006	0.12		0.018
Median	2.9	0.47	1.5	0.023	0.0059	1.8	0.091	25	1.8	0.068	0.075	0.015	1.4	0.008	0.075		0.080
Place (Number of Specimens and Morphological Part) *	**Nb**	**Ni**	**Pb**	**Rb**	**Sb**	**Sn**	**Sr**	**Ta**	**Th**	**Ti**	**Tl**	**U**	**V**	**W**	**Zn**	**Zr**	**Ʃ** **REEs**
(1) Coastal Landscape Park (15; c)	0.031	1.6	0.36	250	0.012	0.15	0.37	0.0041	0.0076	21	0.052	0.0071	1.3	0.012	99	1.2	0.20
(1) Coastal Landscape Park (15; s)	0.041	1.3	0.51	180	0.015	0.15	0.44	0.012	0.016	37	0.14	0.012	1.4	0.016	80	1.2	0.43
(1) Coastal Landscape Park (15; w)	0.036	1.4	0.43	210	0.013	0.15	0.40	0.008	0.012	29	0.096	0.0095	1.3	0.014	89	1.2	0.31
(2) TLP, Osowa (10; w)	0.0053	2.0	1.8	190	0.011	0.075	0.34	0.0007	0.0024	8.1	0.088	0.0027	0.77	0.014	91	0.11	0.12
(X) Mazury, Giżycko (15; c)	0.016	2.0	0.72	290	0.026	0.15	0.55	0.013	0.0090	15	0.052	0.0055	1.4	0.043	87	0.33	0.35
(X) Mazury, Giżycko (15; s)	0.033	1.4	0.81	120	0.022	0.14	0.65	0.016	0.011	15	0.14	0.0066	1.5	0.036	45	0.73	0.48
(X) Mazury, Giżycko (15; w)	0.024	1.7	0.76	200	0.024	0.14	0.60	0.015	0.010	15	0.096	0.0060	1.4	0.039	66	0.53	0.47
(3) Augustów Primeval Forest (16; w)	0.034	2.5	1.3	160	0.057	0.43	0.77	0.034	0.026	22	0.13	0.0071	1.9	0.14	110	0.71	0.76
(4) Pomerania, Szczecinek (22; w)	0.021	1.5	0.82	190	0.045	0.42	0.39	0.035	0.0081	20	0.089	0.0049	2.2	0.16	110	0.99	0.34
(5) Greater Poland, Porażyn (13; w)	0.0098	1.3	1.1	120	0.026	0.26	0.20	0.022	0.0039	11	0.096	0.0037	1.6	0.084	69	0.44	0.17
(6) WLP, Kościerzyna (45; w)	0.017	1.4	1.1	210	0.025	0.23	0.51	0.034	0.0088	16	0.13	0.0053	1.7	0.061	86	0.70	0.25
(7) Tuchola Pinewoods, Osiek (15; w)	0.024	1.7	0.63	200	0.022	0.18	0.46	0.022	0.010	20	0.069	0.0071	1.7	0.062	95	1.0	0.40
(8) Kujawy region, Toruń forests (16; w)	0.039	1.5	0.79	160	0.029	0.20	0.83	0.022	0.021	22	0.16	0.010	1.6	0.082	83	2.1	0.56
(9) Warmia, Morąg (30; w)	0.15	1.7	1.5	230	0.015	0.15	2.0	0.031	0.070	57	0.061	0.028	1.9	0.058	91	5.0	1.8
(10) Warmia, Olsztyn (19; w)	0.041	1.3	1.2	180	0.017	0.14	0.44	0.018	0.013	20	0.082	0.0073	1.5	0.033	80	1.2	0.31
(11) Warmia, Puchałowo (15; w)	0.018	1.6	0.79	230	0.030	0.25	0.52	0.019	0.0076	16	0.094	0.0045	1.6	0.18	130	0.21	0.23
(12) Mazury, Piska Forest (15; c)	0.010	1.5	0.41	240	0.013	0.10	0.23	0.013	0.0027	11	0.051	0.0032	1.2	0.024	98	0.083	0.074
(12) Mazury, Piska Forest (15; s)	0.0064	0.86	0.35	110	0.012	0.13	0.30	0.013	0.0018	5.9	0.14	0.0025	1.4	0.032	39	0.12	0.10
(12) Mazury, Piska Forest (15; w)	0.0082	1.2	0.38	170	0.012	0.11	0.26	0.013	0.0022	8.4	0.095	0.0028	1.3	0.028	68	0.10	0.086
(13) Sudety Mts., Kłodzka Dale (15; c)	0.014	1.9	1.5	580	0.029	0.15	0.57	0.014	0.0071	13	0.16	0.032	1.3	0.033	110	0.29	0.19
(13) Sudety Mts., Kłodzka Dale (15; s)	0.023	1.5	1.7	270	0.020	0.10	1.2	0.011	0.013	12	0.40	0.025	1.2	0.011	54	0.49	0.28
(13) Sudety Mts., Kłodzka Dale (15; w)	0.018	1.7	1.6	420	0.024	0.12	0.88	0.012	0.010	12	0.28	0.028	1.2	0.022	82	0.39	0.23
(Y) Tatra Mountains (12; c)	0.0092	1.3	1.1	150	0.042	0.25	0.30	0.021	0.049	12	0.026	0.038	1.7	0.082	94	0.22	0.13
Mean caps (5 composites)	0.016	1.7	0.82	300	0.024	0.16	0.40	0.013	0.015	14	0.068	0.017	1.4	0.039	98	0.42	0.19
SD	0.009	0.3	0.48	160	0.012	0.05	0.16	0.006	0.019	4	0.052	0.016	0.2	0.027	8	0.44	0.10
Median	0.014	1.6	0.72	250	0.026	0.15	0.37	0.013	0.008	13	0.052	0.0071	1.3	0.033	98	0.29	0.19
Mean stipes (4 composites)	0.026	1.3	0.84	170	0.017	0.13	0.65	0.013	0.010	17	0.20	0.011	1.4	0.024	54	0.53	0.34
SD	0.013	0.2	0.52	64	0.004	0.02	0.34	0.002	0.001	12	0.11	0.008	0.1	0.010	16	0.39	0.15
Median	0.028	1.3	0.66	150	0.017	0.13	0.54	0.012	0.012	13	0.14	0.009	1.4	0.024	49	0.61	0.36
Mean whole (14 composites)	0.032	1.6	1.0	200	0.025	0.20	0.61	0.020	0.014	20	0.11	0.0091	1.5	0.070	89	1.1	0.43
SD	0.036	0.3	0.4	69	0.012	0.11	0.45	0.010	0.017	12	0.05	0.0083	0.3	0.054	18	1.3	0.43
Median	0.022	1.5	0.96	190	0.024	0.16	0.48	0.020	0.010	18	0.095	0.0065	1.6	0.059	87	0.70	0.31

Notes: *, c, s, w (caps, stipes, whole fruitbodies, respectively), TLP (Tricity Landscape Park), WLP (Wdzydze Landscape Park).

## Data Availability

Not applicable.
